# Prevalence and determinants of stunting and wasting among public primary school children in Gondar town, northwest, Ethiopia

**DOI:** 10.1186/s12887-019-1572-x

**Published:** 2019-06-25

**Authors:** Zegeye Getaneh, Mulugeta Melku, Mekuanint Geta, Tadele Melak, Melkamu Tamir Hunegnaw

**Affiliations:** 1 Department of Hematology and Immunohematology, School of Biomedical and Laboratory Sciences, College of Medicine and Health Sciences, University of Gondar, Gondar, Ethiopia; 2 Department of Medical Microbiology, School of Biomedical and Laboratory Sciences, College of Medicine and Health Science, University of Gondar, Gondar, Ethiopia; 3 Department of Clinical Chemistry, School of Biomedical and Laboratory Sciences, College of Medicine and Health Science, University of Gondar, Gondar, Ethiopia; 4 Department of Human Nutrition, Institute of Public Health, College of Medicine and Health Science, University of Gondar, Gondar, Ethiopia

**Keywords:** Nutritional status, Associated factor, Prevalence, School age children

## Abstract

**Background:**

Undernutrition among school age children has an impact on their health, cognition, and educational achievement. Therefore, this study aimed to assess the prevalence and associated factors of stunting and wasting among school age children in Gondar town, northwest, Ethiopia.

**Methods:**

An institution-based cross-sectional study was done among school children aged 6–14 years. Data on socio-demographic, nutritional and dietary status of children were collected using structured questionnaire. Anthropometric measurements were carried out to determine the status of stunting and wasting. Data were entered into Epi-Info version 3.5.3 and transferred to SPSS version 20 for further analysis. Bivariable and multivariable logistic regression models were fitted to identify associated factors of stunting and wasting. Both crude odds and adjusted odds ratios with 95% CI were used to measure the strength of associations. In the multivariable analysis, variables with < 0.05 *p*-values were considered statistically significant.

**Results:**

A total of 523 school age children were with the median age of 12 (10–13 inter quartile range) years participated in the study. The overall prevalence of stunting and wasting among primary school children was 241(46.1%; 95% CI: 42.3, 50.3) and 47 (9%; 95% CI: 6.7, 11.7), respectively. Child age (AOR = 1.9, 95% CI: 1.29, 2.80), public tab/yard water source (AOR = 2.22; 95%CI: 1.46, 3.39), DDS < 4 (AOR = 1.89 95%CI: 1.08, 3.30), tea drinking habit (AOR **=** 0.46, 95%CI: 0.27, 0.80) and anemia (AOR = 1.72 95%CI: 1.05, 2.83) were significant predictors of stunting. Moreover, child age (AOR = 3.91; 95% CI: 1.62, 9.44), maternal/care-givers’ age ≤ 34 (AOR **=** 0.34; 95%CI: 0.16, 0.71), maternal education (AOR = 2.55; 95%CI: 1.15, 5.65), family poverty (AOR = 3.23; 95% CI: 1.30, 7.93) and alcohol consumption (AOR = 2.93; 95%CI: 1.16, 7.42) were found significantly associated with wasting.

**Conclusion:**

Stunting and wasting were then major problems among school age children. Child age, water source for dinking, DDS < 4 and anemia resulted in stunting. On the other hand, child age, maternal education and age, family poverty and alcohol drinking were risk factors for wasting. Therefore, launching community based nutritional education programs, implementing school feeding and strengthening economic level of the communities are essential to reduce the problems.

**Electronic supplementary material:**

The online version of this article (10.1186/s12887-019-1572-x) contains supplementary material, which is available to authorized users.

## Background

Manifested in the form of stunting and wasting, malnutrition is a felt global burden that results in serious public health risks and economic costs [1–4].

Malnutrition among school age children (SAC) in developing nations, especially in Africa, has been linked with morbidity, hygienic practices, dietary intakes and family socioeconomic status [5–7]. It was estimated that nearly 870 million children, 852 million (15% of the population) of whom lived in developing countries suffered from undernutrition between 2010 and 2012 [8, 9]. It is responsible for the death of one-third (7.6 million) of the children on the globe every year [10]. Out of such children, about 178 million were stunted. Of these, around 90% lived in 36 resource limited countries, like Ethiopia [11]. The prevalence of stunting and wasting in Ethiopia ranges from 8.9% [6] to 42.7% [5] and 8.0% [12] to 26.1% [13, 14], respectively.

Primary school age is characterized by dynamic physical growth, mental development and high vulnerability stage [15]. Nutritional deficiencies in SAC are a public health concern especially in resource limited countries. Stunting and wasting which have serious consequences on survival, health, and the development of SAC most commonly affect children in low and middle-income countries. In such countries, around 52.0% of SAC are stunted [16]. According to the Global Education Monitoring report, more than a quarter of children under the age of 15 years living in Sub-Saharan Africa (SSA) were underweight [17]. In 2017, nearly two out of the five stunted children lived in South Asia, while more than one in three lived in SSA, and more than half of all wasted or acutely malnourished children in south Asia, and about one quarter in SSA [18].

Malnutrition begins at pre-school period and may progress into school age. If left untreated, it may have significant negative effects on the academic performance and general well-being of SAC [19, 20].Undernutrition in this stage is the common cause of low school enrolment, high absenteeism, early dropout, unsatisfactory classroom performance and poor general well-being, resulting in poor educational attainment and low intellectual and physical abilities in adulthood [20, 21]. Undernutrition also causes poor school attendance, reduced intelligence quotient, and increased morbidity [4].

Stunting reflects the cumulative effect of problems in socioeconomic status and recurrent infections. It results from long-term nutritional deprivation, inadequate childcare and poor environmental and socio-cultural conditions, poor educational achievement and reduced capital and social progress [11].For these reasons, wide variations in the prevalence of stunting have been observed in different countries. The magnitude of stunting among SAC ranges from 20 to 80% in the world [22]. On the other hand, wasting indicates acute undernutrition, usually due to inadequate food intake or a high incidence of infectious diseases [23, 24]. It was most prominent among people in South-East Asia and Africa, whereas it was generally below 10% in Latin America [25].

Although the factors of stunting and wasting among SAC varies from country to country, child parental education, maternal nutritional knowledge, age, gender and father’s occupation are considered as important risk factors [26, 27]. Studies also show that parasitic infection [13, 28] and family size [13, 22] may contribute to SAC undernutrition.

The reason for conducting this study was to contribute to the goals of increasing the enrolment of children in primary education with the aim of achieving the Sustainable Development Goal (SDG) of universal primary education. Moreover, the lack of data on the health of SAC, growing interest of governments and development agencies in the link between health and education, and the need for a national school health policy for Ethiopia were the other motives for conducting this study. Studies addressing undernutrition have been mostly restricted to under-5 children, rather than focusing school-goes children to assess the magnitude of the problem. Therefore, this study aimed to assess the prevalence and associated factors of stunting and wasting among primary school children in the study area.

## Methods

### Study design, setting and population

An institution-based cross-sectional study was conducted among public primary SAC aged 6–14 years in Gondar town, northwest Ethiopia, from February to May 2017. The town is located in the northern part of the Amhara regional state, northwest Ethiopia. It 727 km from Addis Ababa, the capital of Ethiopia, and had 15,175 SAC going to 26 public primary schools in the 2016/2017 academic year [23].

### Sample size determination and sampling technique

To determine the sample size, a single population proportion formula was used by considering the following assumptions: maximum acceptable error 5, 31% proportion of undernutrition in SAC [29], Z statistic of 1.96, and estimated non-response rate 10%, with a design effect of 1.5, yielding a final sample size was 543.

A systematic random sampling technique was used to select participants. Seven of the public primary schools in the town, were selected by using the lottery method, and participants were assigned to the selected schools proportionally based on their student population. The K value was calculated by dividing the total number of students in each section by the number of children included in the study. The first student was selected randomly between one and K using the lottery method, and the next ones were chosen in accordance with K values until the sample size was achieved. After the students were identified, parents contacted through school directors, children and health extension workers provided the information required.

### Data collection tools

The questionnaire was prepared based on the national survey and modified according to the literature [30]. The questionnaire was initially prepared in English and translated into Amharic, the local language and retranslated to English to maintain consistency. A structured questionnaire was used to collect data on socio-demographic characteristics, household food security status (HFSS), child feeding practices, food consumption patterns, dietary diversity scores (DDS) and health conditions of children. HFSS was assessed by using the standardized questionnaire developed by the Food and Nutritional Technical Assistance (FANTA) [31]. Food consumption patterns and the DDS of the children were assessed using the modified version of the Helen Keller International Food Frequency Questionnaire (FFQ) and a 24-h dietary recall, respectively [32, 33] (Additional file 1).

### Data collection procedures

#### Socio-demographic data

A structured questionnaire was used to collect data on socio-demographic characteristics (child sex and age, age of mother, parents’ education and occupation, marital status of mothers/guardians, family size, home environment and household items), child illness in the last fortnight, deworming within the last 2 months and environmental health condition (water supply, sanitation and housing conditions) from child-parent pairs by face-to-face interviews. Wealth index was determined using the Principal Component Analysis (PCA) [34]. Variables coded between 0 and 1 were entered and analysed using PCA, while those variables with greater than 0.5 communality values were used to produce factor scores which were summed and ranked into tertiles as “poor”, “medium” and “rich”.

#### Household food insecurity assessment

The HFSS was assessed by using a nine item standard questionnaire developed by FANTA [31]. This tool is validated and used in developing countries [35]. A household is considered food secure when it takes took less than 2 of the 27 food insecurity indicators. The response options of the HHFS questionnaire were; “never”, “rarely”, “sometimes”, and “often”. The food secure households were considered when the respondents replied that “never” or “rarely” worried them that their households would not have enough food. On the other hand, mildly food insecure households were defined when the households sometimes or often worried about not having enough food and/or were unable to eat favorite foods, and/ or rarely ate a more monotonous diet than desired. Households that reported they rarely or sometimes ate more monotonous diets than desired sometimes or often, and/or had started to cut back on quantity by reducing the size of meals or number of meals were coded as moderately food insecure [36].

#### Dietary assessment

A modified version of the Helen Keller International FFQ and a 24-h dietary recall were used to determine food consumption patterns and DDS, respectively [32, 33]. A food frequency questionnaire that contains food items that were commonly consumed in the study area were grouped into 10, as cereals, legumes, meat, egg, vegetables, fruits, dairy products, fish and sea foods, sweet foods made with sugar, honey, oil, fat, or butter and any other foods, such as condiments. Children who had DDS ≥ 7, 4–6, and ≤ 3 from the 10 food groups were categorized as high, medium and low dietary diversity, respectively. In addition, tea, coffee and alcohol consumption habits were also assessed by asking about the frequency of regular, daily, and weekly consumptions.

#### Assessment of anthropometric variables

Age, weight, and height of children were recorded according to WHO guideline [37] to generate anthropometric variables. The height of each child was measured in Frankfurt position (head, shoulder, buttocks, knee, and heals touch the vertical board) to the nearest 0.1 cm using a Tanita HR-200 stadiometer. Height was measured without shoes and in a standing position. Body weight was measured to the nearest 0.1 kg without shoes while wearing minimal clothes using a Tanita BWB 800 weighing digital electronic scale. Height and weight measurements were taken in triplicates and the average values were recorded. The weighting scale was calibrated and checked daily using the standard calibration weight of 2 kg iron bars. Then, the two commonly used anthropometric indices, height-for-age (HAZ) and body mass index-for-age (BAZ) Z scores were computed to assess growth and nutritional status of SAC [37]. The Z scores for these nutritional indicators were determined using the WHO Anthro Plus 1.0.4 software program. Stunting and wasting among children were defined as HAZ, and BAZ less than − 2 Z scores, respectively.

#### Hemoglobin and parasitological diagnosis

Hemoglobin (Hb) concentration was measured from a finger-prick blood sample using a HemoCue hemoglobin meter (HemoCue301+, Angelholm, Sweden). The hemoglobin meter was checked daily using control blood samples before the survey started. Two to three drops of blood, enough to fill a cuvette, was obtained from each child using the finger prick method by a professional phlebotomist [38] to measure the Hb level of each participant and reported in grams per deciliter. Approximately, 5 g of fresh stool samples were collected from each individual, following the standard operating procedures (SOPs) using clean and labeled leak-proof stool cups. A 10% formalin was added to the stool specimens and transported in screw-capped cups to the laboratory. Direct saline wet mount and formal-ether concentration methods were used to detect intestinal helminths and protozoa infections microscopically within 2–8 h of sample collection.

### Data quality control

Training was given to data collectors, laboratory technologists and supervisors for 1 day on the objective and relevance of the study, confidentiality of information, participant rights, pre-test, consent, and techniques of interview. They were also trained on how to record laboratory results on result sheets prepared for the purpose. A pre-test was administered on 5% [30] of the sample size out of study schools to ensure the validity of the questionnaire. SOPs and manufacturers’ instructions were strictly followed for all laboratory activities. All laboratory reagents were checked for expiry dates, and laboratory results were recorded on standard report formats using participant identification numbers. Anthropometric measurement, whose mean values were used for analysis were taken three times, the scale was periodically checked using standard weights to ensure accuracy.

#### Statistical analysis

Data were checked, cleaned, and entered into Epi-Info version 3.5.3 and transferred to SPSS version 20 software for further analysis. Anthropometric data of weight and height were converted to height for age Z scores (HAZ) and BMI for age Z scores (BAZ) using the WHO Anthro Plus version 1.4.1. Descriptive statistical data were summarized using texts, tables and figures. A bivariable logistic regression model was fitted to identify factors associated with stunting and wasting. Variables with < 0.2 *p*-values in the bivariate analysis were fitted into the multivariable logistic regression analysis. Both crude (COR) and adjusted odds ratios (AOR) with the corresponding 95% confidence interval (CI) were calculated to show the strength of associations. In the multivariable analysis, variables with < 0.05 *p*-values were considered statistically significant.

#### Ethics approval and consent to participate

Ethical clearance was obtained from the University of Gondar, College of Medicine and Health Sciences and the School of Biomedical and Laboratory Sciences Research and Ethical Review Committees. Permission was given by Gondar town Health and Education Offices, and school authorities. Written informed consent was obtained from parents or guardians after awareness creation activities. Moreover, assent was obtained from children who were more than 7 years old.

## Results

### Socio-demographic characteristics

A total of 523 SAC between 6 and 14 years of age with a 96.3% response rate participated in the study. The median age of the SAC was 12 years, with an inter quartile-range of 10–13 years. Almost half, 269 (51.4%), of the participants were male, 354 (67.7%) were11–14 years old. About 230 (44.0%) and 364 (69.6%) of the mothers were uneducated and housewives, respectively. The majority of the SAC, 414 (79.2%), lived in food secure households; 171 (32.7) were from poor families; only 29 and 30% of the fathers and mothers of attend primary schools, respectively (Table 1).

**Table 1:** Socio-demographic characteristics of public primary school children aged 6–14 years in Gondar town, northwest Ethiopia, 2017

Variables	Category	Frequency	Percentage
Child age (years)	6–10	169	32.3
	11–14	354	67.7
Gender	Male	269	51.4
	Female	254	48.6
Children grade level	1–4	261	49.9
	5–8	262	50.1
Family size	< 6	322	61.6
	≥6	201	38.4
Guardian relationship	Mother	424	81.1
	Father	46	8.8
	Relatives	53	10.1
Marital status of the mothers/guardians	Unmarried	20	3.8
	Married or living together	379	72.5
	Divorced/separated/widowed	124	23.7
Maternal age	≤ 34	259	49.5
	35–44	171	32.7
	≥ 45	93	17.8
Maternal education	No formal education	230	44.0
	Primary	160	30.6
	Secondary and above	133	25.4
Maternal occupation	Housewife	364	69.6
	Government employee	51	9.8
	Private	108	20.7
Paternal education	No formal education	214	40.9
	Primary	144	27.5
	Secondary and above	165	31.5
Paternal occupation	Farmer	73	14.0
	Daily laborer	197	37.7
	Government employee	131	25.0
	Merchant	122	23.3
Wealth status	Low	171	32.7
	Medium	190	36.3
	High	162	33.0
Child illness in the last 2 weeks	Yes	100	19.1
	No	423	80.8
Intestinal parasites	Yes	132	25.2
	No	391	74.8

#### Prevalence of stunting and wasting

The overall prevalence of stunting, wasting and both stunted and wasted (overlap) among SAC were 241(46.1, 95% CI: 42.3, 50.3), 47 (9, 95%CI: 6.7, 11.7) and 21 (4.0, 95% CI: 2.5, 5.7), respectively**.** Out of the total 241 stunted SAC, 124 (51.5%), 178 (73.9%) and 119 (49.4%) were male, 11–14 years old and 5–8 the grade pupils, respectively. Greater than 46% of the male (*n* = 269) participants were stunted. Of the total 47 wasted SAC, 27 (57.4%), 40 (85.1%) and 31 (66.0%) were male, 11–14 years old and 5–8 the grade learners, respectively. The Hb level of the participants ranged from 9.7 g/dl to 16.3 g/dl with a mean (± SD) value of 12.72 ± 1.08 g/dl, and 81 (15.5%) of the participants were anemic. Of the anemic SAC, 45 (55.5%) and 7 (8.6%) were stunted and wasted, respectively. Out of a total the 132 (25.2%) SAC infected with at least one of the seven species of parasites (*Entamoeba histolytica*, *H*.*nana*, *Giardia lamblia*, *Schistosoma mansoni*, *Ascaris)*, 20.7% (*n* = 50) and 23.4% (*n* = 11) were found stunted and wasted, respectively. Of the total 126 SAC consuming non-piped water at home, 76 (31.5%) were stunted.

#### Stunting and wasting by food insecurity and food diversity score

Of the children in food secure households, about 48.3 and 8.2%were stunted and wasted, respectively. On the other hand, 42.2 and 12.8% of the participants from food in HHs were stunted and wasted, respectively. Of the total participants, 68 (13.0%) had food less than 4 diversity score, and of these 60.3 and 11.8% were stunted and wasted, respectively (Table 2).

**Table 2:** Stunting and wasting by household food security and dietary diversity score among public primary school children in Gondar town, northwest Ethiopia

Characteristics	Stunting		Wasting		
	Stunted number (%)	Non-stunted number (%)	Wasted number (%)	Non-wasted number (%)	Total
Household food security					
Food secured	195 (48.3)	219 (51.7)	33(8.2)	381(81.8)	404 (100)
Mild food insecured	25(37.9)	41(62.1)	10(15.2)	56(84.8)	66 (100)
Moderate food insecured	12(41.4)	17(58.6)	2(6.9)	27(93.1)	29 (100)
Severe food insecured	9(64.3)	5(35.7)	2(14.3)	12(85.7)	14 (100)
Food diversity score					
DDS^a^ < 4	41(60.3)	27(39.7)	8(11.8)	60(88.5)	68 (100)
DDS 4–6	83(47.2)	93(52.8)	13(7.4)	163(92.6)	176 (100)
DDS ≥ 7	117(41.9)	162(58.1)	26(9.3)	253(90.7)	279 (100)

^a^*DDS* Dietary diversity score

### Factors associated with stunting and wasting

In the bivariable logistic regression model, age of a child, water source, DDS, anemia, tea, coffee and alcohol drinking habits, and deworming were independent predictors of stunting with a *p*-value of less than 0.2. Therefore, all of the variables were fitted into a multivariable logistic regression model. However, only child age, DDS, water source, tea taking habits and anemia remained significantly associated with stunting. In this study, children aged 11–14 yearswere1.9 times more likely to be stunted (AOR = 1.9; 95% CI: 1.29, 2.80) compared to children aged 6–10 years. Less than 4 DDS, tab/yard water source, and anemia were 1.89, 2.22 and 1.72 times more likely to cause stunting, respectively, than cases otherwise (Table 3).

**Table 3:** Bivariable and multivariable logistic regression analysis of stunting among SAC in Gondar town public primary schools, northwest Ethiopia, 2017 (*n* = 523)

Variable		Stunting			COR (95% CI)	AOR (95%CI)	*P*-value
		Stunted	Non-Stunted	Total			
Child’s Age	6–10	63 (37%)	106 (62.7%)	169 (100)	1	1	0.001
	11–14	178 (50.3%)	176 (49.7%)	354 (100)	1.70 (1.170, 2.476)	1.90 (1.29, 2.80) *	
Dietary diversity score	< 4	41(60.3%)	27(39.7%)	68 (100)	2.10(1.22, 3.61)	1.89 (1.08, 3.30)*	
	4–6	83 (47.2%)	93 (52.8%)	176 (100)	1.24 (0.85,1.81)	1.13 (0.77, 1.67)	
	≥7	117 (41.9%)	162 (58.1%)	279 (100)	1	1	
Water source	Piped into dwelling	160(42.0%)	221 (58.0%)	381 (100)	1	1	0.0001
	Non-piped water	76(60.3%)	50(39.7%)	126 (100)	2.10 (1.39, 3.17)	2.22 (1.46, 3.39)*	
	Tanker water	5(31.2%)	11(68.8%)	16 (100)	0.63(0.21, 1.84)	0.47 (0.1, 1.42)	
Coffee drinking	Yes	70(52.6%)	63(47.4%)	133 (100)	1.42(0.96, 2.11)	1.00 (0.6, 1.61)	0.991
	No	171(43.8%)	219(56.2%)	390 (100)	1	1	
Tea drinking habit	Yes	41 (62.1%)	25 (37.9%)	66 (100)	0.48 (0.28, 0.81)	0.46 (0.27, 0.80) *	0.006
	No	200 (43.8%)	257(56.2%)	457 (100)	1	1	
Anemia	Non-anemic	196 (44.3%)	246 (55.7%)	442 (100)	1	1	0.032
	Anemic	45 (55.6%)	36 (44.4%)	81 (100)	1.57 (0.97, 2.53)	1.72 (1.05, 2.83) *	
Alcohol consumption	Yes	23(54.8%)	19(45.2%)	42 (100)	1.46 (0.77,0.74)	1.19 (0.61, 2.33)	0.613
	No	218(45.4%)	262(54.6%)	480 (100)	1	1	
Deworming	Yes	163(43.7%)	210(56.3%)	373 (100)	1	1	0.170
	No	78(52.0%)	72(48.0%)	150 (100)	1.40 (0.95, 2.04)	1.32(0.89, 1.96)	

*Indicate statistically significant at *p*-value 0.05

On the other hand, in the bivariable logistic regression model, child age, child grade level, maternal/care-giver age, maternal and paternal education, household food insecurity, wealth index, water source, child illness and alcohol consumption were found significantly associated with wasting at a *P*-value of less than 0.2. In the multivariable logistic regression model, child age, maternal age, maternal education, wealth index and alcohol consumption remained significantly associated with wasting (*P* < 0.05). This study showed that children aged 11–14 years were 3.91 (AOR = 3.91; 95% CI: 1.62, 9.44), children from poor families 3.2 (AOR = 3.2; 95%CI: 1.30, 7.93) and those who a consumed alcohol 2.9 times (AOR = 2.93; 95%CI: 1.16, 7.42) more likely to be wasted (Table 4).

**Table 4:** Bivariable and multivariable logistic regression analysis of wasting among SAC in Gondar town public primary schools, northwest Ethiopia, 2017 (*n* = 523)

Variable		Wasting			COR (95% CI)	AOR (95%CI)	*P*-value
		Wasted	Non-Wasted	Total			
Child’s age	6–10	7 (4.1%)	162 (95.9%)	169 (100)	1		0.002
	11–14	40 (11.3%)	314 (88.7%)	354 (100)	2.95 (1.292, 6.728)	3.91(1.62, 9.44) *	
Grade Label	Grade 1–4	16(6.1%)	245(93.9%)	261 (100)	1	1	0.963
	Grade 5–8	31(11.8%)	231(88.2%)	262 (100)	2.06 (1.095,3.857)	0.98(0.42, 2.28)	
Maternal/ Care-giver Age	≤34	22(8.5%)	237(91.5%)	259 (100)	0.42(0.21,0.82)	0.34 (0.16,0.71)*	0.001
	35–44	8(4.7%)	163(95.3%)	171(100)	0.22(0.09,0.53)	0.17(0.07, 0.42) *	
	≥45	17(18.3%)	76(81.7%)	93 (100)	1	1	
Maternal education	No formal education	12(5.2%)	218(94.8%)	230(100)	1	1	0.031
	Primary education	19(11.9%)	141(88.1%)	160(100)	2.45 (1.15, 5.20)	2.55(1.15, 5.65) *	
	Secondary & above	16(12.0%)	117(88.0%)	132 (100)	2.48 (1.14, 5.43)	2.76 (1.19, 6.38) *	
Father’s education	No formal education	18(8.4%)	196(91.6%)	214(100)	1	1	0.225
	Primary education	8(5.6%)	136(94.4%)	144(100)	0.64 (0.27, 1.52)	0.50 (0.20, 1.28)	
	Secondary& above	21(12.7%)	144(87.3%)	165 (100)	1.59 (0.82, 3.09)	1.10(0.50, 2.42)	
Household food security	Food secured	33(8.0%)	381(92.0%)	414 (100)	1	1	0.637
	Food insecured	14(12.8%)	95(87.2%)	109(100)	1.70(0.88, 3.31)	1.21 (0.56, 2.62)	
Wealth index	Poor	19 (11.1%)	152 (88.9%)	171(100)	2.13 (0.93, 4.85)	3.23(1.30, 7.93) *	0.038
	Medium	19 (10.0%)	171 (90.0%)	190(100)	1.89 (0.83, 4.30)	2.25(0.94, 5.39)	
	Rich	9 (5.6%)	153 (94.4%)	162(100)	1	1	
Water source	Piped into dwelling	34(8.9%)	347 (91.1%)	381 (100)	1	1	0.318
	Public tab/yard water	9(7.1%)	117(92.9%)	126 (100)	0.79 (0.37, 1.69)	0.82 (0.36, 1.86)	
	Protected spring/tanker	4(25.0%)	12 (75.0%)	16 (100)	3.40(1.04, 11.13)	2.55(0.64, 10.08)	
Child Illness	Yes	15(14.2%)	91(85.8%)	106 (100)	1.98(1.03, 3.82)	1.87(0.90, 3.78)	0.082
	No	32(7.7%)	385(92.3%)	417 (100)	1	1	
Alcohol consumption	Yes	8 (19.0%)	34 (81.0%)	42 (100)	2.66 (1.15, 6.14)	2.93(1.16, 7.42)*	0.023
	No	39 (8.1%)	441 (91.9%)	480 (100)	1	1	

*Indicate statistically significant at *p*-value 0.05

## Discussion

Undernutrition among SAC is still a major public health problem in developing countries, including Ethiopia. The magnitude of stunting and wasting in this study was 46.1 and 9%, respectively. The prevalence was a high burden that might result in many complications in terms of academic achievement, physical and mental development, and health related outcomes of the SAC. The prevalence of stunting was especially, high implying that a large number of SAC were suffering from chronic undernutrition.

The prevalence of stunting among SAC in this study (46.1%) was comparable with the results of other similar studies conducted in Durbete town, northwest Ethiopia (42.7%) [39], Fogera and Libo Kemkem districts, both northwest Ethiopia (42.7%) [5] and Arba Minch, Southern Ethiopia (41.9%) [12]. However, this finding was higher than those of studies done in Dale woreda, Southern Ethiopia (35.9%) [13], Addis Ababa, Ethiopia (19.6%) [29],Tigray, northern Ethiopia (28.5%) [14], and Gondar town, northwest Ethiopia (23%) [40]. This discrepancy might be due to the existence of a high prevalence of chronic childhood stunting in the Amhara region as reported by 2011 and 2016 Ethiopian DHS [34, 41]. This can be explained by the fact that children stunted early in life remain short because the problem is an irreversible phenomenon [42].

Besides, the prevalence of stunting was higher in the study area than what was reported in Sri Lanka (26.9%) [43], Kenya (24.5%) [7], Burkina Faso (29.4%) [44], Cambodia (40.4%) [44], India (18.5%) [15], and New Guinea (38.9%) [45]. However, our finding was much lower than those of studies conducted in Bangladesh (60%) [22] and the Volta region of Ghana (50.3%) [46]. This inconsistency might be due to the variability of risk factors in different geographic regions plus socioeconomic status and dietary diversity of SAC.

In the current study, the risk factors for stunting were child age, DDS, water source, tea taking habits and anemia. Older (11–14 years) SAC were significantly associated with stunting. This was in line with other findings [12, 14, 24, 44, 47]. The odds of being stunted were two times higher among older (11–14 years) SAC compared to 6–10 years. This might be due to the fact that a high prevalence of childhood stunting that reflects undernutrition starts in the first years of life [48] and becomes worse at different phases of growth and results in short adult stature [49].

Moreover, SAC with < 4 DDS had a greater chance of being stunted. This was consistent with the result of a study done in Dagoretti, Kenya [7]. SAC who took less than four varieties of food had a higher risk of being stunted than those who took four and more varieties of food. Thus, the odds of stunting was 1.9 times higher among SAC who had < 4 DDS than their counterparts. This might be due to the fact that cereal-based monotone diet with poor quality, quantity, and frequency of feeding that doesn’t fulfill micronutrient requirements, such as iron, vitamin B12, folate and other essentials requirements for child growth [50].

In our study, anemia was found significantly associated with stunting among SAC. Anemic children were two times more likely to be stunted than non-anemic ones. This result was in agreement with those of other studies in Asia and Africa [24, 29, 44].

Similarly, the prevalence of stunting was high among SAC who used non-piped water. This was in line with that of a study conducted in Dhaka city, Bangladesh [22]. Children born in houses with no access to clean water were at risk for diseases which ultimately increased the risk of malnutrition. There is a clear evidence of the association between drinking water source and diarrhoeal morbidity among children [51]. This revealed that children born in houses with no access to clean water were at risk for diseases which ultimately increased the risk of malnutrition. Unclean water supply has a direct relation to infectious diseases, especially diarrhea. Repeated or persistent diarrhea due to unsafe water supply indicates the nutritional status of SAC. Children suffering from diarrhoea do not benefit fully from food because frequent stools prevent adequate absorption of nutrients [52, 53].

Children who had tea taking habits were 0.46 times less likely to be stunted than their counterparts. The association of tea drinking with stunting was not reported by other authors; so we are unable to make comparisons in the present study. The possible reason for this might be the fact that tea contains antioxidants (phenols and polyphenols like catechins, the aflavins, flavenoids and epicatechins) which prevent a huge host of diseases and disorders (cardiovascular and Parkinson diseases and several kinds of cancers) [54] which might decrease the probability of getting stunted.

The prevalence of wasting (9%) among SAC in this study was comparable with the results of other similar studies in Arba Minch (8.0%) [12], New Guinea (8.3%) [45], Burkina Faso (11.2%) [45] and south-eastern Iran (8.8%) [55]. But our finding was lower than those of studies done in Dale woreda, southern Ethiopia (14%) [13], Fogera and Libo Kemkem districts, northwest Ethiopia (20.8%) [5], India (33.3%) [15], and the Volta region of Ghana (19.4%) [46]. The possible reason for this might be socio-economic differences and the variability of risk factors across different geographic settings.

In our study, child and maternal age, maternal education, wealth index and alcohol consumption were found statistically significant factors for SAC wasting. The probability of child wasting increases with age. This finding is in line with that of a study in Fogera and Libo Kemkem districts, Ethiopia [5]. The possible explanation for this is that when children mature, household socioeconomic characteristics may change in conjunction with behavioral and biological variables as important risk factors. However, this finding is different from that of a study done in northern Ethiopia [14].

In the present study, wasting was higher among SAC from the lower socioeconomic class (low wealth index) than those of the upper class. This was in line with the finding in Aksum town, northern Ethiopia [56]. The reason might be that coming from a lower income family inhibits the ability to eat healthfully and affects the type of health care service a child could receive due to lack of affordable services. This could lead to an inability to diagnose micronutrient deficiencies, including iron deficiency and malnutrition in children in general [57].

School age children whose mothers had no formal education were 2.5 times less likely to be thin compared to those whose mothers had secondary and above education. This is dissimilar from that of a study done in Dibrugarh, India [58]. The reason for this might be that o non-educated mothers are more likely to stay at home and have a better chance to feed their children more frequently, while educated mothers have more chances to be employed and spend their times away from home. This increases their work load and reduces the time they have to spend with their children, thereby affecting biological qualities like motherhood, reproduction, lactation and child rearing, etc. [59]. Therefore, the nutritional status of the children of employed mothers is often poorer than that of unemployed ones [60]. All of these can lead to SAC wasting.

In this work, children with alcohol drinking habits at home were 2.9 times more likely to be wasted compared to their counterparts. This finding was supported by study done in Cape town, South Africa [61]. The possible explanation for this is that alcohol use may result in a more significant reduction in dietary intake with energy from alcohol not compensating for the total loss of dietary energy intake. This causes inadequate energy and nutrient intake that could manifest itself in the adolescent as wasting. In other words, taking alcohol inhibits the growth hormone of humans [62]. More importantly, alcohol contributes to malnutrition by replacing and impairing essential nutrient absorption by damaging the cell lining of the stomach and the intestine. They also lead to malnutrition by interfering with absorption, storage or metabolism and the utilization of essential nutrients. Besides, alcohol inhibits the breakdown of nutrients into usable molecules by decreasing the secretion of digestive enzymes from the pancreas [63, 64].

The odds of wasting was lower for children who lived with younger mothers/caregivers compared to children living with older mothers/caregivers. This was consistent with the study conducted in Hula, southern Ethiopia [65]. What could be because older mothers had less health seeking behaviors compared to younger ones. Malnutrition was lowest among children born to mothers in their mid-late twenties. Mothers in their late 30’s were more likely have children suffering from malnutrition [66].

The major limitation of this study is that the micronutrient status of children other than that of anemia was not measured, and its real association with stunting and wasting was not known. The other limitations are that subclinical infections other than intestinal parasites were not assessed, limiting the generalizability of the findings. Besides, as the study is cross-sectional, no causal link can be inferred. Moreover, the investigation is limited to public primary schools only.

## Conclusion

In conclusion, stunting and wasting were common problems among SAC in this study. This indicates that early intervention is vital and calls for health programs that aim at reducing childhood stunting and wasting, especially in the first 2 years of life, and the prenatal period as stunting among school children is the result of damage that starts in utero and early childhood. Factors leading to stunting were child age, source of potable water, DDS < 4 and anemia. On the other hand, child age, maternal education and age, family poverty, and alcohol drinking were risk factors for wasting. Therefore, initiating a community-based nutrition education program, implementing school feeding and strengthening the economic status of the community are essential strategies to reduce the high burdens of stunting and wasting among SAC. Stunting is a major public health concern. Therefore, this study recommends that school health and nutrition programs be implemented to improve the nutritional status of SAC in the study area.

## Additional file

Additional file 1: Questionnaire. (DOC 218 kb)

## Data Availability

All data generated or analysed during this study are available from the corresponding author, so that it can be obtained based up on reasonable request.

## Abbreviation

AOR: Adjusted odds ratio
BAZ: Body Mass Index-for-Age Z score
CI: Confidence interval
DDS: Dietary diversity score
FANTA: Food and Nutritional Technical Assistance
FFQ: Food Frequency Questionnaire
HAZ: Height-for-Age Score
Hb: Hemoglobin
HFSS: Household food security status
IQR: Inter quartile range
MDG: Millennium Development Goal
OR: Crudes odds ratio
PCA: Principal Component Analysis
SAC: School age children
WHO: World health organization
