# Supplementary material for: Prevalence and determinants of stunting and wasting among public primary school children in Gondar town, northwest, Ethiopia
Source: BMC Pediatr. 2019 Jun 25;19:207. doi: 10.1186/s12887-019-1572-x (PMC6591879; doi:10.1186/s12887-019-1572-x)
Supplement: Supplementary file 1 — Questionnaire. (DOC 218 kb) [file 12887_2019_1572_MOESM1_ESM.doc]

***English version questionnaire***

**Information sheet and household head and individual consent form**

**Information sheet**

Good morning? / Good afternoon? My name is --------------- I am the member of research team from University of Gondar. We are conducting a study about the anemia status of school-age children. Your child and your household are chosen by chance to participate in the study. We need to collect data about socio-demography; food consumed in the household, anemia and stool test will be done on your children. I want to assure you that all of your answers will be strictly confidential. You have the right to participate or not, to stop the interview at any time or to skip any questions that you don’t want to answer. Your participation is completely voluntary but your experiences could be very helpful to design better anemia prevention and treatment strategy for school-age children in the town. We will take a few drops of your children capillary blood to measure anemia, a match head size of stool to test intestinal parasite and we will inform you right away the result. We will follow a standard aseptic procedure to run the entire laboratory test. The interview may take approximately half an hour to complete.

Do you have any questions?

**Consent form**

**Purpose:** The purpose of this research is to study “prevalence of anemia and associated risk factors among school age children in Gondar town primary schools, Northwest Ethiopia.”

**Benefits:** There is no direct benefit from participating in this research but the results of the study are no doubt to be important for improvement of risk management related with anemia.

**Risks:** By participating in this research project, there will be minimum pain while I take a capillary blood from your finger tip for a few minutes, dedication of their time by respondents.

**Incentives:** There is no any payment to be gained by taking part in this research.

**Confidentiality**: Every piece of information will be kept confidentially. Information will be accessed by the research only and no wastage will be allowed.

**Contact persons:** If you want to know more information, have any question, you can contact the committee through the researchers address below.

Principal investigator-:

Zegeye Getaneh: 0910107078 Email= [zegeyegetaneh91@gmial.com](mailto:zegeyegetaneh91@gmial.com) Posta: 196

Advisors:

Mr. Mulugeta Melku: 0910084521

Mr. Fikir Asrie: 0918031782

I undersigned have been informed that the questionnaire is conducted to gather information about “prevalence of anemia and associated factors among school age children in Gondar administrative town, Northwest Ethiopia.” The result of the study will help the government and health facilities involved in this service provision to design intervention program as well as for the proper management of anemia related complications. I also agreed about the confidentiality of the responses to be at a highest possible level.

Signature:-___________________ Date _______________________________

Do you agree to be interviewed? And do you agree your children to be tested for anemia?

Respondents agree to be interviewed?

Yes No

May I begin the interview now?

To be signed by interviewer

I certify that I have read the above consent procedure to the participant.

Signed: _____________________

**CHILD ASSENT FORM**

I am---------------- from University of Gondar. I am doing a study to figure out prevalence and associated factors of Anemia in school age children in Gondar town primary schools. We are asking you to take part in the research study because you are chosen by chance to participate in the study. We are asking you to take part in the research study because your participation is very helpful to design better anemia prevention and treatment strategy for school-age children in the town. For this research, we will take a few drops capillary blood to measure anemia, approximately 5 gram of stool to test intestinal parasite and we will inform you right away the result. We will follow a standard aseptic procedure to run the entire laboratory test.

I want to assure you that all of your answers will be kept strictly. You have the right to participate or not, to stop the interview at any time or to skip any questions that you don’t want to answer. Your participation is completely voluntary but your experiences could be very helpful to design better anemia prevention and treatment strategy for school-age children in the town. The interview may take approximately half an hour to complete.

You should know that:

- You do not have to be in this study if you do not want to.
- You may stop being in the study at any time. (If there is a question you don’t want to answer, just leave it blank.)
- Your parent(s)/guardian(s) were asked if it is OK for you to be in this study. Even if they say it’s OK, it is still your choice whether or not to take part.
- You can ask any questions you have, now or later. If you think of a question later, you or your parents can contact me at (provide contact information for researcher(s), and advisor if graduate student).

Sign this form only if you have understood what you will be doing for this study, have had all your questions answered, have talked to your parent(s)/legal guardian about this project, and agree to take part in this research

Your Signature ___________ Printed Name ___________________ Date __________

Name of Parent(s) or Legal Guardian(s) _______________________________

Researcher/explaining study Signature _________Printed Name _____________Date________

| **Interview information**  ⮚ Name of the kebele ________________Name of the village________________  ⮚ Name of the school____________  ⮚ Questionnaire IDN……………………………|___|___|___|  ⮚ Household IDN …………………………………|___|___|___|  ⮚ Date of interview |___|___| Day |___|___| Month l___|___|___|___| |Year  Time started |___|___| Hour |___|___| Minutes  Time ended |___|___| Hour |___|___| Minutes  Result * |___|  ⮚ Interviewer Name ______________________________________  ⮚ Supervisor ______________________________________  ⮚ Checked by ______________________________________  ⮚ Entered by 1) ____________________________________  2) ____________________________________  *Result codes:  1=completed 4=refused 7=other (specify)  2=not available 5=partly completed  3=postponed 6=incapacitated ___________________ |
| --- |

**QUESTIONNAIRE**

| **Part one**  **A. Socio-demographic characteristics of the child and household head**  *I would like to ask you a few questions about you and your child* | | | | | | | | | | | | | | | | | | |
| --- | --- | --- | --- | --- | --- | --- | --- | --- | --- | --- | --- | --- | --- | --- | --- | --- | --- | --- |
| S.No | Characteristics or questions | Answers and codes | | | | | | | | | | | | | | | Skip ption | |
| 101 | What is your relationship with the child? | Mother.......................1 Father......................2  Step father ……..3 Step mother ……4  Brother………….5 Sister…………….6  Relative………7 Others(Specify)…….99_______ | | | | | | | | | | | | | | |  | |
| 102 | Residence | Rural…… 1 Urban ……2 semi-Urban …..3 | | | | | | | | | | | | | | |  | |
| 103 | How old are you?  *(Age of the primary care giver*  *completed years)* | Age __________________ | | | | | | | | | | | | | | |  | |
| 104 | What is your religion? | Orthodox Christian.......1 Muslim..............2  Protestant ...............3 Jeush .................4  Other (specify) ..................9999__________________ | | | | | | | | | | | | | | |  | |
| 105 | To which ethnic group do you belong? | Amhara..........1 Oromo........2 Tigrie…3 Kimant-------4  Other (specify) ………99__________________ | | | | | | | | | | | | | | |  | |
| 106 | What is your current Marital status? | Never marred --------------1 Married -----------------2  Living together----------3 Separated---------------4  Divorced-------------5 Widowed-------------6 | | | | | | | | | | | | | | |  | |
| 107 | What is your educational status?  ***(Care giver/ mother)*** | Primary (1-8) .............1 Secondary (9-12) ........2  College/university .......3 Read and write.............4  Illiterate …………….. 5 | | | | | | | | | | | | | | |  | |
| 108 | What is your occupation?  *(****Care giver/ mother)*** | Farmer and housewife.....1 Housewife..................2  employee/private ............3 Student........................4  Merchant.........................5 Local drink seller.......6  Commercial sex worker....7 Maid servant............8  Daily laborer................9 Unemployed............10  Other (specify) ................99 | | | | | | | | | | | | | | |  | |
| 109 | What is child’s father or care giver educational status?  *(****Care giver/ father)*** | Primary (1-8) .............1 Secondary (9-12) ........2  College/university .......3 Read and write.............4  Illiterate …………….. 5 | | | | | | | | | | | | | | |  | |
| 110 | What is the child’s father occupation?  *(****Care giver/ father****)* | Farmer……1 Employee of government/private…2  Student ………3 Merchant…………………..4  Daily laborer….5 Unemployed …………….6  Other (specify)..........99_____________________ | | | | | | | | | | | | | | |  | |
| 111 | How many are the total number of family members currently living in this house?  ***(including all individuals will stay***  ***and stayed for greater than 6***  ***month)*** | _____________________________ | | | | | | | | | | | | | | |  | |
| **B. Information pertaining to household environment, which is about housing characteristics and household possessions**  *Now I will ask you some questions about your* *housing characteristics and household possessions* | | | | | | | | | | | | | | | | | | |
| 112 | Will you please describe your family’s household living structure? | Private ………..1 Kebele……..2  Rent………3 Relatives’………4  other (specify)________________________________ 99 | | | | | | | | | | | | | | |  | |
| 113 | Main construction material used in exterior walls:  circle all that apply | Wood with mud.....1 Bricks.....................2  Tin ........................3 Stone with mud...........4  Card board ...........5 Stone with lime/cement ......6  other (specify) .................................99 | | | | | | | | | | | | | | |  | |
| 114 | What is main construction material used for the roof?  ***circle all that apply*** | thatch/leaf/mud ..........................1  rustic mat/plastic sheet ...............2  reed/bamboo ................................3  wood planks ................................4  cardboard.....................................5  corrugated iron/metal ..................6  wood ...........................................7  asbestos/cement fiber ..................8  cement/concrete ..........................9  roofing/shingles ...........................10  other (specify) .................................99 | | | | | | | | | | | | | | |  | |
| 115 | Main construction material used for the floor:  *circle all that apply* | earth /sand .................................1  dung ...........................................2  wood planks ................................3  polished wood or parquet ............4  vinyl or asphalt strips .................5  ceramic tiles ................................6  cement ........................................7  carpet ...........................................8  other (specify) .................................99 | | | | | | | | | | | | | | |  | |
| 116 | How many Bed rooms do you use? | _______________________ | | | | | | | | | | | | | | |  | |
| 117 | Among the following which property do you have in your home?  ***(interviewer: circle all that apply)*** | **Yes—1** | | | | | | | | | | **No--2** | | | | |  | |
|  | Electricity | **1** | | | | | | | | | | **0** | | | | |  |  |
|  | Watch/clock | **1** | | | | | | | | | | **0** | | | | |  | |
|  | Radio | **1** | | | | | | | | | | **0** | | | | |  |  |
|  | Television | **1** | | | | | | | | | | **0** | | | | |  |  |
|  | Mobile Telephone | **1** | | | | | | | | | | **0** | | | | |  |  |
|  | House Phone | **1** | | | | | | | | | | **0** | | | | |  |  |
|  | Refrigerator | **1** | | | | | | | | | | **0** | | | | |  |  |
|  | Chair | **1** | | | | | | | | | | **0** | | | | |  |  |
|  | A bed with cotton/Sponge mattres | **1** | | | | | | | | | | **0** | | | | |  |  |
|  | Electric Mitad | **1** | | | | | | | | | | **0** | | | | |  |  |
|  | Kerosene Lamp/pressure | **1** | | | | | | | | | | **0** | | | | |  |  |
|  | Solar light | **1** | | | | | | | | | | **0** | | | | |  |  |
|  | None | **1** | | | | | | | | | | **0** | | | | |  |  |
| 118 | What type of fuel does your household mainly use for cooking?  ***(interviewer: allow multiple***  ***answers)*** | Electricity ........1 Natural gas........2  Biogas............... 3 Kerosene............4  Wood............... 5 Charcoal ............6  Straw/shrubs/grass........ 7  Other (specify).................................99 | | | | | | | | | | | | | | |  | |
| 119 | Does any member of the household own the following? [interviewer: circle all that apply | **Yes** | | | | | | **No** | | | | | | | | |  | |
|  | bicycle | 1 | | | | | | **0** | | | | | | | | |  |  |
|  | motorcycle/scooter | 1 | | | | | | **0** | | | | | | | | |  |  |
|  | animal drawn cart | 1 | | | | | | **0** | | | | | | | | |  |  |
|  | car/truck | 1 | | | | | | **0** | | | | | | | | |  |  |
| **Part two: Household food security status**  *Now I will ask you about the food security of your house based on the last four weeks* | | | | | | | | | | | | | | | | | | |
|  | Characteristics or questions | | | | Answers and codes | | | | | | | | | | | | Skip option | |
| **201** | In the past four weeks, did you worry that your household would not have enough food? | | | | Yes………………………….1  No…………………………2 | | | | | | | | | | | | If yes go to 203 | |
| **202** | How often did this happen in the past four weeks? | | | | Rarely (once or twice/ month) ……1  Sometimes (3-10 times / month) …2  Often (>10 times/ month)…………3 | | | | | | | | | | | |  | |
| **203** | In the past four weeks, were you or any household member not able to eat the kinds of foods you preferred because of a lack of resources? | | | | Yes……………...............................1  No………………………………2 | | | | | | | | | | | | If yes go to  205 | |
| **204** | How often did this happen in the past four weeks? | | | | Rarely (once or twice/ month) ……1  Sometimes (3-10 times / month) …2  Often (>10 times/ month)……… 3 | | | | | | | | | | | |  | |
| **205** | In the past four weeks, did you or any household member have to eat a limited variety of foods due to a lack of resources? | | | | Yes…….........................................1  No……………………………….2 | | | | | | | | | | | | If yes go to  207 | |
| **206** | How often did this happen in the past four weeks? | | | | Rarely (once or twice/ month) ……….1  Sometimes (3-10 times / month) ….2  Often (>10 times/ month) ………….3 | | | | | | | | | | | |  | |
| **207** | In the past four weeks, did you or  Any household member have to eat some foods that you really did not want to eat because of a lack of resources to obtain other types of food? | | | | Yes……………………1  No……………………….2 | | | | | | | | | | | | If yes go to  209 | |
| **208** | How often did this happen in the past four weeks? | | | | Rarely (once or twice/ month) ……….1  Sometimes (3-10 times / month) ……….2  Often (>10 times/ month)……………….3 | | | | | | | | | | | |  | |
| **209** | In the past four weeks, did you or any household member have to eat a smaller meal than you felt you needed because there was not enough food? | | | | Yes……...........................................1  No……………………………2 | | | | | | | | | | | | If yes go to  211 | |
| **210** | How often did this happen in the past four weeks? | | | | Rarely (once or twice/ month) ……….1  Sometimes (3-10 times / month) …….2  Often (>10 times/ month)……………3 | | | | | | | | | | | |  | |
| **211** | In the past four weeks, did you or any other household member have less frequent meals because there was not enough food? | | | | Yes……….......................................1  No……………………………….2 | | | | | | | | | | | | If yes go to 213 | |
| **212** | How often did this happen in the past four weeks? | | | | Rarely (once or twice/ month) …1  Sometimes (3-10 times / month) …2  Often (>10 times/ month)………3 | | | | | | | | | | | |  | |
| **213** | In the past four weeks, was there ever no food to eat of any kind in your household because of lack of resources to get food? | | | | Yes…………………….1  No………………………….2 | | | | | | | | | | | | If yes go to  215 | |
| **214** | How often did this happen in the past four weeks? | | | | Rarely (once or twice/ month) ……1  Sometimes (3-10 times / month) …2  Often (>10 times/ month)………3 | | | | | | | | | | | |  | |
| **215** | In the past four weeks, did you or any household member go to sleep at night hungry because there was not enough food? | | | | Yes……………………..1  No………………………2 | | | | | | | | | | | | If yes go to 217 | |
| **216** | How often did this happen in the past four weeks? | | | | Rarely (once or twice/ month) ……1  Sometimes (3-10 times / month) …2  Often (>10 times/ month)…………3 | | | | | | | | | | | |  | |
| **217** | In the past four weeks, did you or any household member go a whole day and night without eating anything because there was not enough food? | | | | Yes …………..1  No………………………………2 | | | | | | | | | | | |  | |
| **218** | How often did this happen in the past four weeks? | | | | Rarely (once or twice/ month) ……1  Sometimes (3-10 times / month) …2  Often (>10 times/ month)…………3 | | | | | | | | | | | |  | |
| **Part three**  **Household dietary diversity score**  Now I would like to ask you about the types of foods that you or anyone else in your household *ate yesterday during the day and at night* either separately or combined with other foods. | | | | | | | | | | | | | | | | | | |
|  | **Food items** | | | | | **30 days Frequency of consumption** | | | | | | | | | | | | |
|  | Please chose type of foods and how many times you used | | | | | **Per Month** | | | | **Per Week** | | | | **Per day** | | | | |
|  |  |  |  |  |  | Never | Once | | 2-3 | 1-2 | 3-4 | | 5-6 | 1-2 | 2-3 | 4-5 | | >6 |
| 301 | **Cereal food** | | | | |  | | | | | | | | | | | | |
|  | Corn/maize, rice, wheat, sorghum, teff, millet or any other grains or foods made from these (e.g. bread, porridge, injera, pasta, macaroni, rice) | | | | |  |  | |  |  |  | |  |  |  |  | |  |
| 302 | Roots and tubers | | | | |  |  | |  |  |  | |  |  |  |  | |  |
|  | Any potatoes, bulla, or any other food made from roots or tubers? | | | | |  |  | |  |  |  | |  |  |  |  | |  |
| 303 | **Vegetable** | | | | |  |  | |  |  |  | |  |  |  |  | |  |
|  | Dark green leafy vegetables cassava, kale, spinach Other vegetables (e.g. tomato, onion, garlic, cabbage, lettuce and sweet potato that are orange inside, red sweet pepper, banana and 100% fruit juice made from these | | | | |  |  | |  |  |  | |  |  |  |  | |  |
| 304 | **Fruits** | | | | |  |  | |  |  |  | |  |  |  |  | |  |
|  | vitamin A rich fruits like pumpkin, carrot, squash, mango, papaya, peach, and other fruits avocado, lemon, grapes orange 100% fruit juice made from these | | | | |  |  | |  |  |  | |  |  |  |  | |  |
| 305 | Flesh meat and organ meat | | | | |  |  | |  |  |  | |  |  |  |  | |  |
|  | Beef, lamb, goat, chicken, liver, kidney, heart or other organ meats or blood-based foods | | | | |  |  | |  |  |  | |  |  |  |  | |  |
| 306 | Eggs- eggs from chicken, duck, or any other egg | | | | |  |  | |  |  |  | |  |  |  |  | |  |
| 307 | Fish and sea foods - fresh or dried fish or shellfish | | | | |  |  | |  |  |  | |  |  |  |  | |  |
| 308 | Legumes, nuts and seeds | | | | |  |  | |  |  |  | |  |  |  |  | |  |
|  | Dried beans, dried peas, lentils, nuts, seeds or foods made from these | | | | |  |  | |  |  |  | |  |  |  |  | |  |
| 309 | Milk and milk products | | | | |  |  | |  |  |  | |  |  |  |  | |  |
|  | Milk, cheese, yogurt or other milk products | | | | |  |  | |  |  |  | |  |  |  |  | |  |
| 310 | Oils and fats | | | | |  |  | |  |  |  | |  |  |  |  | |  |
|  | Any food made by adding oil, fats or butter added to food or used for cooking | | | | |  |  | |  |  |  | |  |  |  |  | |  |
| 311 | Any other foods, such as condiments, coffee, tea, Sweet drink | | | | |  |  | |  |  |  | |  |  |  |  | |  |
| **Part Four**  **Socio-demographic and Information pertaining to past history and current illness of the child**  *Now I will ask you about the child past history and current illness* | | | | | | | | | | | | | | | | | | |
| 1. **Child’s socio-demographic characteristics** | | | | | | | | | | | | | | | | | | |
| 401 | Sex of the child | | | 1. Male 2. Female | | | | | | | | | | | | |  | |
| 402 | When he/she was borne (date of birth)? | | | ___/____/_____(DD/MM/YY) | | | | | | | | | | | | |  | |
| 403 | Age of the child incompleted months | | | ___________ | | | | | | | | | | | | |  | |
| 404 | Current Grade level of the child | | | ____________ | | | | | | | | | | | | |  | |
| **B. Information pertaining to past history and current illness of the child** | | | | | | | | | | | | | | | | | | |
| 405 | Does the child took iron tablet this week? | | Yes-------1 No---------2  Unknown----------98 | | | | | | | | | | | | | |  | |
| 406 | Does the child took vitamin A in the last 6 months? | | Yes-------1 No---------2  Unknown----------98 | | | | | | | | | | | | | |  | |
| 407 | Does the child took anti-parasite drugs in the last 6 months? | | Yes ……….1 No --.2  Don’t know………………98 | | | | | | | | | | | | | |  | |
| 408 | Does the child sick with any disease in the past 2 weeks? | | Yes-------1 No---------2  Unknown----------98 | | | | | | | | | | | | | | 413 | |
| 409 | Does the child had fever in the past 2 weeks? | | Yes-------1 No---------2  Unknown----------98 | | | | | | | | | | | | | |  | |
| 410 | Does the child had cough in the past 2 weeks? | | Yes-------1 No---------2  Unknown----------98 | | | | | | | | | | | | | |  | |
| 411 | Does the child had diarrhea in the past 2 weeks? | | Yes-------1 No---------2  Unknown----------98 | | | | | | | | | | | | | |  | |
| 412 | Was it bloody diarrhea? | | Yes-------1 No---------2  Unknown----------98 | | | | | | | | | | | | | |  | |
| 413 | Is there any bed net in the house for Malaria prevention? | | Yes-------1 No---------2  Unknown----------98 | | | | | | | | | | | | | |  | |
| 414 | How long you used the bed net? | | Yes-------1 No---------2  Unknown----------98 | | | | | | | | | | | | | |  | |
| 415 | Does the child used the bed net Yesterday? | | Yes-------1 No---------2  Unknown----------98 | | | | | | | | | | | | | |  | |

**24-Hour** Dietary Diversity Questionnaire

| **Dietary Diversity Questionnaire**  Please tell me the foods (meals and snacks) that you ate or drank yesterday during the day and night, whether at home or outside the home. Start with the first food or drink eaten in the morning. Write down all foods and drinks mentioned. When composite dishes are mentioned, ask for the list of ingredients. | | | | | | |
| --- | --- | --- | --- | --- | --- | --- |
| Breakfast | Snack | Lunch | Snack | Dinner | Snack | |
|  |  |  |  |  |  | |
|  |  |  |  |  |  | |
|  |  |  |  |  |  | |
|  |  |  |  |  |  | |
|  |  |  |  |  |  | |
|  |  |  |  |  |  | |
|  |  |  |  |  |  | |
|  |  |  |  |  |  | |
| When the respondent recall is complete, fill in the food groups based on the information recorded above. For any food groups not mentioned, ask the respondent if a food item from this group was consumed | | | | | | |
| s/no | Food group | | Examples | | | Yes=1  No= 0  I don’t know= 99 |
|  | Starchy staples (cereals) | | corn/maize, rice, wheat, sorghum, teff, millet or any other grains or foods made from these (e.g. bread, porridge, injera, pasta, macaroni, rice) | | |  |
|  | White roots and tubers | | white potatoes, white yam, white cassava,  or other foods made from roots | | |  |
|  | Dark green leafy vegetables | | dark green leafy vegetables cassava, kale, spinach | | |  |
|  | vitamin A rich fruits and vegetables | | pumpkin, carrot, squash, or sweet potato that are orange inside, red sweet pepper, mango, papaya, peach, and 100% fruit juice made from | | |  |
|  | Other fruits and vegetables | | other vegetables (e.g. tomato, onion, garlic, cabbage, lettuce and Fruits like avocado, banana, lemon, grapes orange) and 100% fruit juice made from these | | |  |
|  | Organ meat | | liver, kidney, heart or other organ meats or blood-based foods | | |  |
|  | Flesh meat | | beef, lamb, goat, chicken, | | |  |
|  | Fish and sea foods | | fresh or dried fish or shellfish | | |  |
|  | Eggs | | eggs from chicken, duck, or any other egg | | |  |
|  | Legumes, nuts and seeds | | dried beans, dried peas, lentils, nuts, seeds or foods made from these | | |  |
|  | Milk and milk products | | milk, cheese, yogurt or other milk products | | |  |
|  | Oils and fats | | oil, fats or butter added to food or used for cooking | | |  |
|  | Any other foods, such as condiments, coffee, tea, Sweet drink? | | Condiments, coffee, tea, Sweet drink | | |  |

**Thank you very much for giving your precious time and your collaboration!!**
